# Steering teens safe: a randomized trial of a parent-based intervention to improve safe teen driving

**DOI:** 10.1186/1471-2458-14-777

**Published:** 2014-07-31

**Authors:** Corinne Peek-Asa, Joseph E Cavanaugh, Jingzhen Yang, Vidya Chande, Tracy Young, Marizen Ramirez

**Affiliations:** Department of Occupational and Environmental Health, University of Iowa College of Public Health, 105 River St, S143B CPHB, Iowa City, IA 52242 USA; Department of Biostatistics, University of Iowa College of Public Health, Iowa City, IA USA; Nationwide Children’s Hospital, Ohio State University, Columbus, OH USA; Blank Children’s Hospital, Des Moines, IA USA; University of Iowa Injury Prevention Research Center, Iowa City, IA USA; Department of Occupational and Environmental Health, University of Iowa College of Public Health, Iowa City, IA USA

**Keywords:** Road traffic, Driving safety, Adolescent, Parent communication

## Abstract

**Background:**

Crashes are the leading cause of death for teens, and parent-based interventions are a promising approach. We assess the effectiveness of Steering Teens Safe, a parent-focused program to increase safe teen driving.

**Methods:**

Steering Teens Safe aimed to improve parental communication with teens about safe driving using motivational interviewing techniques in conjunction with 19 safe driving lessons. A randomized controlled trial involved 145 parent-teen dyads (70 intervention and 75 control). Intervention parents received a 45-minute session to learn the program with four follow-up phone sessions, a DVD, and a workbook. Control parents received a standard brochure about safe driving. Scores were developed to measure teen-reported quantity and quality of parental communication about safe driving. The main outcome measure was a previously validated Risky Driving Score reported by teens. Because the Score was highly skewed, a generalized linear model based on a gamma distribution was used for analysis.

**Results:**

Intervention teens ranked their parent’s success in talking about driving safety higher than control teens (p = 0.035) and reported that their parents talked about more topics (non-significant difference). The Risky Driving Score was 21% lower in intervention compared to control teens (85% CI = 0.60, 1.00). Interaction between communication quantity and the intervention was examined. Intervention teens who reported more successful communication had a 42% lower Risky Driving Score (95% CI = 0.37, 0.94) than control parents with less successful communication.

**Conclusions:**

This program had a positive although not strong effect, and it may hold the most promise in partnership with other programs, such as Driver’s Education or Graduated Driver’s License policies.

**Trial registration:**

ClinicalTrials.gov NCT01014923. Registered Nov. 16, 2009.

## Background

Motor vehicle crashes are the leading cause of injury and death among US teenagers, and teen drivers have crash rates higher than any other age group [1–3]. Teen driver crashes also cause serious injuries and fatalities to other occupants: approximately 30% of fatal crashes that involve a teen driver lead to death or serious injury to occupants of other vehicles involved in the crash [2]. Compared with adult drivers, a higher percentage of teen fatal crashes involve driver error [4]. Crash rates among novice drivers are highest during the first months of unsupervised driving, and interventions to increase safe teen driving may be most effective if they affect early driving experiences [5, 6].

Existing studies indicate that parents have varying levels of engagement in helping their children mature to independent drivers, and they face challenges in knowing exactly how to best influence safe driving behaviors [7, 8]. Although studies have found that parents and teens believed supervised driving was important, more than a quarter of parents spent less than 25 total hours driving with their teen [9, 10]. Furthermore, parents spent most of their supervised driving time in the daylight, on residential roads, and in light traffic -- the least challenging driving environment -- and did not supervise driving in more challenging environments over time [11].

Studies have found that parents generally exhibit poor monitoring and control of risky driving behaviors. Conversations about driving focus more on basic vehicle operations (e.g. changing a tire) and defined rules (such as when the teen can use the car and when to be home) rather than conversations that set expectations and motivate teens to act safely (e.g. wearing their seat belts or avoiding distractions) [12–14]. Studies have found that when safety was discussed, topics were very general, such as instructing teens to “know the rules of the road.” Yang et al. found that teens who reported that their parents talked about safety had more safety-prone attitudes, but what parents themselves reported talking about was not related to safety attitudes – indicating that it is not so much what the parent says but what the teen actually hears that influences safety attitudes [15]. This limited evidence clearly indicates that the quantity and quality of conversations as well as the topics addressed are all critical in influencing safe driving behavior.

Studies of parenting behavior show that good parenting practice can have profound effects on adolescent development and are strongly tied to reduced risk-taking behaviors [16–18]. A recent study has suggested that parenting skills can be successfully taught to and implemented by parents and caregivers [7]. Based on this growing evidence, the Community Preventive Services Task Force has recommended parent/caregiver interventions that use person-to-person techniques to improve parenting skills as an evidence-based approach [19].

We present results from a randomized controlled trial of an intervention called Steering Teens Safe that taught parents communication strategies to encourage safe driving. The intervention group was compared to a control group to measure teen-reported quantity and quality of safe driving communication and self-reported risky driving behaviors.

## Methods

### Study setting and participants

Parents and teens were recruited from eight high schools located in and around Des Moines and Iowa City, Iowa. Additional recruitment occurred among parents employed in two hospitals located in these same cities. Recruitment began in 2007 and was completed in 2010. Teens who were at least 15 years of age and anticipated applying for an intermediate driver’s license were eligible. In Iowa, the intermediate license is the first opportunity for unsupervised driving, and the intervention was timed to occur three months prior to intermediate licensure. Parents and teens had to speak English, and a parent was eligible to enroll only once (in the case of multiple eligible teenaged children during the study period).

### Recruitment and randomization

Using passive recruitment methods, information about the study was mailed to potentially eligible parents identified through the participating schools and to all employees of the hospitals. Interested participants provided their contact information to study coordinators through a return postcard or by calling a number provided in the letter. The research team followed up to screen for eligibility and, for those eligible, a meeting by phone or in person was scheduled to collect parent consent, teen assent, and conduct random assignment. A binary random number generator was used to assign participants to the intervention and control groups. This study was approved by the University of Iowa and the Blank Children’s Hospital Human Subject Protection Committees.

### Intervention arm

The intervention, called Steering Teens Safe, was designed to help parents positively motivate their teen to make safe driving decisions by providing safe driving discussion topics and effective communication strategies. Parents received a workbook that identified nineteen safety lessons divided into four topics: Basic safety principles (take driving seriously, seat belt use, distraction, impaired driving, being a safe passenger); Safe Driving Skills (traffic signals, safe speeds, changing lanes, following too closely, communicating with other vehicles, and turning); Rural Driving (2-lane roads, gravel roads, uncontrolled intersections, trucks and farm equipment), and Special Situations (bad weather, animals, emergency vehicles, work zones). Each lesson included talking points and instructed parents to talk about, demonstrate, and supervise their teen in the lesson. These lessons were chosen based on analyses of Iowa Department of Transportation crash data and previous research on teen driving errors. This is one of the first interventions to incorporate safe driving on rural roads.

Techniques from motivational interviewing were taught to parents to help them effectively communicate with their teens. Motivational interviewing encourages active listening to motivate behavior change in others and has been effective in the areas of substance abuse, smoking cessation, and dietary modifications [20–22]. The tools used in this study include the use of open-ended questions, affirmations, reflective listening, summarizing, rolling with resistance, and reframing. The approach has not been widely used in traffic safety but is well-suited for teens making decisions without supervision.

In an initial 45-minute session, parents were taught motivational interviewing techniques to effectively communicate the 19 safety lessons. They were also provided with a DVD demonstrating sample parent-teen conversations and laminated cards summarizing the techniques. Parents received three 30-minute follow-up phone calls to provide additional intervention support and to measure process indicators of implementation. A process evaluation of the intervention indicated that parents found the intervention highly acceptable and did use the study materials [23].

### Control arm

Parents randomized into the control arm received a general safety booklet that is provided by the Iowa Department of Motor Vehicles when new drivers obtain a driver’s permit.

### Study measures

Data for this study are from parent and teen baseline surveys and teen surveys filled out one- and six-months post-licensure. This analysis used teen-reported outcomes, which are the best indicators of the intervention success [15]. Following a logic model developed for the study, we first hypothesized that the intervention would lead to improved quality and quantity of communication about safe driving for intervention compared with control dyads. Communication was measured using a Safe Driving Topic score that measured talking quantity and a Talking Success score that measured talking quality, both measured at the six month post-licensure follow-up survey. The Safe Driving Topic score was based on the 19 topics in Steering Teens Safe. Teens were asked how often their parents talked about each topic on a five-point scale ranging from “never” to “very frequently.” The Safe Driving Topic Score was calculated as the sum of responses for each topic. A high Safe Driving Topic Score was defined as being above the median; a low score as below the median. For the Talking Success Score, each teen was asked to rate the success of the conversations about safe driving on a scale of one to ten, with ten being the most effective. A high Talking Success Score was defined as being 8 or above and a low score as below 8.

We next hypothesized that if parents improved communication about safe driving, teens would have reduced risky driving behaviors. Risky driving was measured through the self-reported Risky Driving Inventory which was adapted to reflect the specific driving goals of the intervention [24]. Risky driving was reported at one- and six-months post-licensure. Respondents reported the number of times in the past three weeks that they performed each driving behavior, and an overall score was calculated as the sum of risky driving behaviors. The original version of the index showed high reliability, with a Cronbach’s coefficient alpha for responses of 0.88 at baseline and 0.81 at a 3-month follow-up measurement.

### Sample size

**Figure 1:**
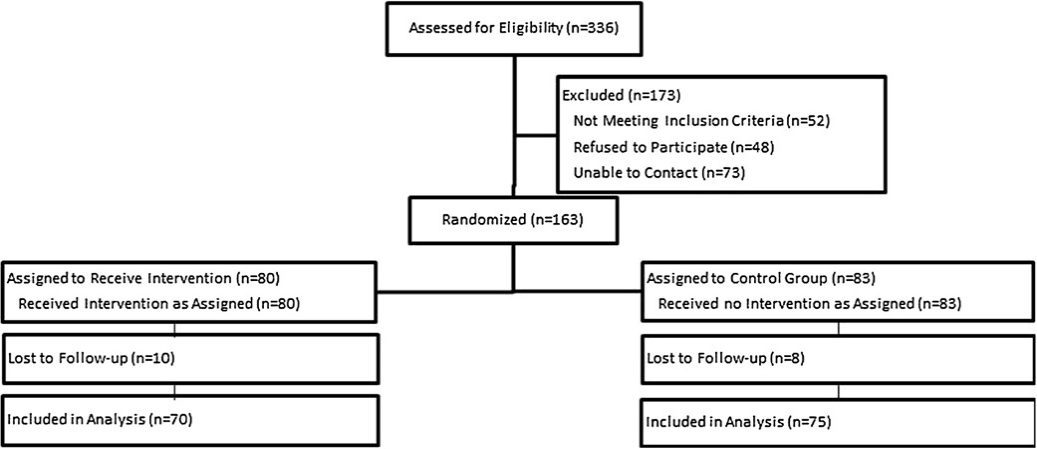
**CONSORT diagram.**

The proposed sample size to achieve 90% power to detect a risky driving score difference of 20% was 176 parent/teen dyads with 88 in each study arm. Using our passive enrollment protocol, a total of 336 potential participants provided contact information for follow-up (Figure 1). Of these, 52 were ineligible (mostly due to delayed licensure), 48 refused, and we were unable to contact 73; the remaining 163 (48.5%) parent/teen dyads consented/assented to enroll in the study. The final sample size, based on teen one- and six-month follow-up surveys analyzed here, was 145, with 70 (84.5% follow-up) in the intervention arm and 75 (93.8% follow-up) in the control arm (Figure 1).

### Data analysis

Data analysis was conducted using SAS version 9.3. Chi-square tests were used to compare distributions of qualitative parent and teen characteristics between the intervention and control groups. The Talking Topics Score and Talking Success Score were compared between intervention and control groups using t-tests.

The Risky Driving Score, the dependent variable, was highly skewed, and a log transformation did not result in approximate normality. Generalized linear models based on a gamma distribution and a log link function was found to best fit the data. To accommodate the repeated measures collected from the teen at one- and six-months post licensure, the models were fit using generalized estimating equations with an exchangeable working correlation structure. Each model included a main effect for group membership (intervention/control) and for survey period (one and six months).

Risky Driving Scores between intervention and control teens were compared for the two groups overall. Interaction between the intervention and quality and quantity of parent communication was examined by stratifying on intervention status and communication status. Because we did not reach recruitment goals to achieve 90% power, we examined confidence intervals for the overall intervention using 95%, 90% and 85% levels, and to examine the precision of the confidence limits we assessed upper-to-lower confidence limit ratios [25, 26].

## Results

Baseline demographic characteristics did not differ significantly by intervention status, indicating that randomization was successful (Table 1). Over 80% of the parents were mothers, while sex of the participating teen was evenly divided between males and females. Father/daughter dyads were the least common dyad combination. The majority of parents were in their 40’s, married, and two-thirds were college graduates. The majority of participating teens were 15, as older teens were only eligible if they had delayed licensure. Over two-thirds began supervised driving at age 14 (which is the youngest age of legal supervised driving in Iowa), although 17.9% of intervention teens and 23.9% of control teens started driving younger than age 14. More than 70% of teens reported driving 6 or more days a week at the beginning of the study.

**Table 1:** **Demographic characteristics for parent/teen dyads by intervention status**

Parent characteristics	Intervention n (%)	Control n (%)	p-value
Gender^a^			0.597
Father/Daughter	6 (8.6)	3 (4.0)	
Father/Son	7 (10.0)	8 (10.7)	
Mother/Daughter	29 (41.4)	37 (49.3)	
Mother/Son	28 (40.0)	27 (36.0)	
Age			0.234
30-39	7 (10.0)	14 (18.9)	
40-49	49 (70.0)	50 (67.6)	
50-59	14 (20.0)	10 (13.5)	
Marital status			0.363
Married	60 (85.7)	60 (80.0)	
Divorced/Separated/Widowed/Single	10 (14.3)	15 (20.0)	
Race^a^			0.393
White	68 (98.6)	73 (97.3)	
Non-white	1 (1.4)	2 (2.7)	
Education			0.874
Some college or lower	23 (33.3)	27 (36.5)	
Four-year college graduate	28 (40.6)	27 (36.5)	
Some graduate school or higher	18 (26.1)	20 (27.0)	
Employment			0.387
Employed full-time	53 (77.9)	53 (71.6)	
Employed part-time/other	15 (22.1)	21 (28.4)	
Teen characteristics			
Gender			0.688
Male	35 (50.0)	35 (46.7)	
Female	35 (50.0)	40 (53.3)	
Age^a^			
15	64 (92.8)	71 (94.7)	
16-17	5 (7.2)	4 (5.3)	
Grade^a^			0.708
9^th^	11 (15.7)	9 (12.0)	
10^th^	54 (77.1)	62 (82.7)	
11^th^ and 12^th^	5 (7.1)	4 (5.3)	
Race^a^			0.175
White	67 (98.5)	70 (94.6)	
Non-white	1 (1.5)	4 (5.4)	
Age at first drive			0.805
12 or younger	5 (7.5)	6 (8.4)	
13	7 (10.4)	11 (15.5)	
14	49 (73.1)	47 (66.2)	
15 or older	6 (9.0)	7 (9.9)	
Frequency of driving per week (on average; at 6-month follow-up)^a^			0.390
Less than 1 day a week	0 (0.0)	1 (1.3)	
1 to 2 days a week	4 (5.7)	5 (6.7)	
3 to 5 days a week	17 (24.3)	11 (14.7)	
6 or more days a week	49 (70.0)	58 (77.3)	

^a^Exact tests were conducted due to small count.

As the first step in evaluating the intervention, we compared the quantity and quality of conversations about safe driving as reported by intervention and control teens (Table 2). Avoiding distractions when driving was the most frequently addressed topic for both intervention and control teens; driving in bad weather, wearing a seat belt, and maintaining a safe speed were also among the most frequently addressed topics. Driving on rural roads, including 2-lane roads, gravel roads, uncontrolled intersections, and sharing the road with farm equipment were the least frequently addressed topics, although these were more frequently discussed by intervention parents. Although the average Talking Topics Score, which is the sum of the scores for individual topics, was higher for the intervention group, this difference was not statistically significant. The mean overall success score, ranked from one through ten, was significantly higher for intervention (7.97) than for control teens (7.32, p = 0.035).

**Table 2:** **Teen-reported talking topics score and talking success score**

Driving topics ^a^	Intervention (n = 70) mean (SD)	Control (n = 75) mean (SD)	p-value ^b^
Basic safety principles			
Take the job of driving seriously.	3.60 (1.08)	3.60 (1.09)	1.000
Always wear your seat belt	3.77 (1.35)	3.67 (1.36)	0.643
Avoid distractions while driving	3.94 (0.83)	3.91 (1.00)	0.814
Never drive after drinking or using drugs	3.51 (1.54)	3.37 (1.50)	0.577
Be a safe passenger	3.44 (1.26)	3.17 (1.26)	0.199
Important skills for safe driving			
Follow all traffic signals	3.43 (1.27)	3.49 (1.20)	0.753
Maintain a safe speed	3.64 (1.20)	3.80 (1.12)	0.416
Changing lanes	2.77 (1.24)	2.61 (1.04)	0.406
Don’t follow too closely	2.84 (1.25)	2.96 (1.13)	0.554
Communicate with other vehicles	2.54 (1.11)	2.63 (1.12)	0.653
Turning at intersections	2.61 (1.18)	2.57 (1.09)	0.805
Special skills for rural roads			
Driving on 2-lane roads	2.59 (1.22)	2.47 (1.05)	0.553
Driving on gravel roads	2.83 (1.31)	2.75 (1.28)	0.704
Uncontrolled intersection	2.67 (1.30)	2.56 (1.18)	0.590
Share the road with trucks and farm equipment	2.60 (1.26)	2.42 (1.78)	0.390
Other roadway situations			
Collisions with animals	3.14 (1.22)	2.80 (1.25)	0.097
Driving in bad weather	3.81 (1.01)	3.71 (1.14)	0.549
Emergency maneuvers	2.87 (1.28)	2.61 (1.17)	0.208
Other special driving situations	2.81 (1.28)	2.69 (1.20)	0.546
Talking topics score	59.44 (17.72)	57.63 (15.51)	0.512
Talking success score	7.97 (1.44)	7.32 (2.16)	0.035

^a^1 = Never, 2 = Rarely, 3 = Sometimes, 4 = Frequently, 5 = Very frequently.

^b^p-values based on t-test.

We next evaluated the intervention by comparing teen-reported risky driving behavior by intervention status. Intervention teens had lower mean Risky Driving Scores than control teens at both one (4.49 for intervention; 5.68 for control) and six months (6.26 for intervention; 7.91 for control) (Table 3). Risky driving scores generally increased from one to six months. Intervention and control teens were first compared without regard to the success of the intervention. The generalized linear model indicated that the intervention was associated with a 21% lower mean Risky Driving Score (Estimated mean ratio = 0.79, 95% CI = 0.55, 1.15). The intent-to-treat mean ratio was significant based on the 85% interval (0.60, 0.99).

**Table 3:** **Intervention effects on risky driving mean scores for teens, by treatment group**

		Risky driving score		Gamma generalized linear model estimates			
	n	One month average (estimated)	Six month average (estimated)	(Estimated mean ratio) ^a^	Confidence level at which statistical significance reached		
					95% CI	90% CI	85% CI
Model 1: Overall groups							
Control	75	5.68	7.91	Ref.	————	————	————
Intervention	70	4.49	6.26	0.79	(0.55, 1.15)	(0.58, 1.08)	(0.60, 0.99)
Upper to lower confidence limit ratio (CLR)					2.1	1.9	1.7
Model 2: Intervention/Talking Topics Score							
Control/Low score	34	6.72	8.94	Ref.	————		
Control/High score^b^	41	4.63	6.15	0.76	(0.49, 1.18)		
Intervention/Low score^b^	34	5.13	6.82	0.69	(0.46, 1.03)		
Intervention/High score^b^	36	3.94	5.24	0.58	(0.36, 0.94)		
Model 3: Intervention/Talking success score							
Control/Low score	63	9.32	4.16	Ref.			
Control/High score^b^	12	5.08	7.63	0.54	(0.27, 1.08)	(0.31, 0.97)	
Intervention/Low score^b^	5	10.51	8.61	1.12	(0.62, 2.05)	(0.68, 1.87)	
Intervention/High score^b^	65	5.31	4.34	0.56	(0.29, 1.09)	(0.32, 0.98)	

^a^Based on a repeated measures model that includes one- and six-month scores; ^b^Upper-to-lower confidence limit ratios were under 2 for Talking Topics Scores and under 3 for Talking Success Scores.

We next examined the effects of the intervention on teen risky driving considering the Talking Topics Score, assuming that both parent communication success and the intervention could influence risky driving. We hypothesized that parents who participated in the intervention and who had more success in communication would have teens with lower risky driving. Compared with control teens with low Safe Driving Topics Scores, intervention teens who had high Safe Driving Topics Scores had a 42% lower Risky Driving Score (95% CI = 0.36, 0.94). Intervention teens who reported low Talking Topics Scores had a 31% lower Risky Driving Score compared with control teens with low scores, but this difference was not statistically significant at even the 0.15 level. Among the control teens, those whose parents had high Talking Topics Scores had a 24% lower Risky Driving Score than those whose parents had a low Talking Topics Score (not significant). Intervention groups with both low and high Talking Topics Scores had lower risky driving scores compared with the control group with a low Talking Topics Score.

Intervention teens who reported a high Talking Success Score had a 44% lower Risky Driving Score (90% CI 0.32, 0.98) when compared with control teens who reported a low Talking Success Score. Control teens who reported a high Talking Success Score had a similar effect as intervention teens, showing a 46% lower Risky Driving Score than control teens who reported a low Talking Success Score. Intervention teens who reported a low Talking Success Score had a 12% higher Risky Driving Score (90% CI 0.68, 1.88). However, only five intervention teens had a low Talking Success Score.

## Discussion

This research contributes to the small but growing body of evidence that parents can positively influence their teen’s driving behavior, and that this influence can persist even after their teen begins to drive unsupervised. Differences between the overall intervention and control groups were marginally significant. However, intervention teens had lower Risky Driving Scores regardless of whether or not the teen reported parent communication scores as high or low. Intervention teens who reported that their parents had high communication scores had the lowest risky driving scores with a 42% reduction (95% CI 0.36, 0.94). Among control teens, those who reported high communication scores had lower risky driving than control teens who reported their parents had low communication scores. These findings indicate that risky driving behavior was successfully reduced among teens whose parents participated in the intervention or were successful communicators.

Our findings indicate that Steering Teens Safe is not likely to have extremely large effects, but the program does have a high probability of having a positive effect. The approach used in Steering Teens Safe may hold the greatest promise in augmenting other strategies to reduce teen driver crashes, in particular those directly focused on the teen. Graduated Driving Licensure laws have been effective in reducing teen driver crashes by limiting driving in the highest-risk situations (such as late-night driving and driving with other teen passengers) during the early phases of licensure. Strong evidence from more than a decade of research estimates that GDL has reduced teen driving injury and fatal crashes by 20 – 40% [27–30]. Recognizing that the elements of GDL laws vary between states and that effectiveness will be moderated by the level of adherence, programs such as Checkpoints have been developed to assist parents in setting driving rules, restrictions, and expectations [31–33]. In particular, parents participating in the Checkpoints program had implemented more restrictions on late-night driving, teen passengers, and high-speed roadways than control parents [34].

These programs have focused primarily on rules and restrictions so that teens begin driving in low-risk environments. Developing safe driving behaviors is difficult to address solely through driving restrictions, especially when teens begin to drive without supervision. Some risk-taking is a normal part of adolescent development, and once teenagers begin to drive unsupervised, they could choose unsafe driving behaviors, such as not using their seat belts, to rebel against restrictions on driving [7, 35]. Furthermore, restrictions are more likely to be followed by teens if they are communicated to them effectively by parents [15]. Thus, approaches that combine restrictions and self-motivation with effective communication are likely to be the most effective.

Research on parenting and teen risk behavior supports this combination. For example, one study found that parent communication alone was not associated with reduced youth substance use, but communication in combination with parental monitoring was [36]. Studies of healthy eating have found that food rules and restrictions are associated with overeating and eating without hunger, but strong parent–child communication bonds are associated with good eating behaviors [37]. Steering Teens Safe focused exclusively on communication strategies between parents and teens and did not integrate rules and restrictions. Future research will integrate these facets into Steering Teens Safe.

Enrollment goals for this study were not met. Recruitment was challenging because we enrolled only parent/teen dyads who both agreed to consent, and because our Human Subject Protection Office required a passive recruitment strategy. Although our enrollment goals were not met, loss to follow-up was very low (15.6% for intervention and 6.3% for control). Because our target sample was not met, we examined multiple confidence limits for the overall program. All of the confidence intervals showed consistent and high precision with the ratio of the upper to lower confidence limits ranging from 1.7 to 2.1. Additionally, the majority of the confidence limits were within the range of showing program effectiveness at all of the different alpha levels examined. For example, although the main effect estimate (0.79) was not significant at an alpha level of 0.05, the confidence limit of 0.55 to 1.15 shows only a small proportion of the limit above the null value of 1.00.

We want to comment on this approach, as we notice increasing reliance on the arbitrary choice of an alpha level of 0.05 for most epidemiological analyses. Interpreting findings based on multiple confidence levels and the interval level of consistency and precision demonstrates the alpha level at which these data meet the general criteria of statistical significance, and examination of confidence limits provides information about the consistency and precision of the estimates. This approach shows that our findings are significant with a lower level of certainty, yet does not diminish their practical importance. With the dearth of parent tools to encourage safe teen driving and research that shows a clear need for such tools [9], interventions that show success with 90% and 85% confidence intervals that are consistent and precise among relatively small sample sizes show some promise.

This study has several additional limitations. Outcomes were self-reported. Teen reports of communication quality and quantity were used instead of parents so that we could measure the intervention as it was received by teens. However, self-report measures of both communication and risky driving could be biased in either direction. A conservative bias could have occurred if teens in the intervention group were more aware of their risky driving behaviors and thus reported more of them. Alternatively, teens in the intervention group may have been more likely to report better communication and reduced risky driving because they perceived those as the desired responses. Future research will integrate more objective outcome measures.

## Conclusion

Based on previously published findings that parents rated this intervention highly and that parents were successful in engaging in the intervention techniques, and with the findings here that suggest a relationship with reduced risky driving behavior, we recommend further development of interventions that increase parent’s quantity and quality of safe driving communication with their teens.
